# Pspace: a program that assesses protein space

**DOI:** 10.1186/1751-0473-2-6

**Published:** 2007-10-23

**Authors:** Mihaly Mezei, Ming-Ming Zhou

**Affiliations:** 1 Department of Structural and Chemical Biology, Mount Sinai School of Medicine, New York University, One Gustave L. Levy Place, New York, New York 10029, USA

## Abstract

**Background:**

We describe a computer program named Pspace designed to a) obtain a reliable basis for the description of three-dimensional structures of a given protein family using homology modeling through selection of an optimal subset of the protein family whose structure would be determined experimentally; and b) aid in the search of orthologs by matching two sets of sequences in three different ways.

**Methods:**

The prioritization is established dynamically as new sequences and new structures are becoming available through ranking proteins by their value in providing structural information about the rest of the family set. The matching can give a list of potential orthologs or it can deduce an overall optimal matching of two sets of sequences.

**Results:**

The various covering strategies and ortholog searches are tested on the bromodomain family.

**Conclusion:**

The possibility of extending this approach to the space of all proteins is discussed.

## Background

Recent advances in comparative structural modeling have demonstrated that reasonably reliable model structures can be built for proteins that share greater than 20–25% sequence similarity to their template proteins whose structures are determined experimentally [1]. As a consequence, Vitkup and colleagues [2] have discussed the possible approaches to obtain three-dimensional structural information of all known proteins based on structures of a judiciously chosen subset of proteins from all protein families that would be experimentally determined.

**Figure 1:**
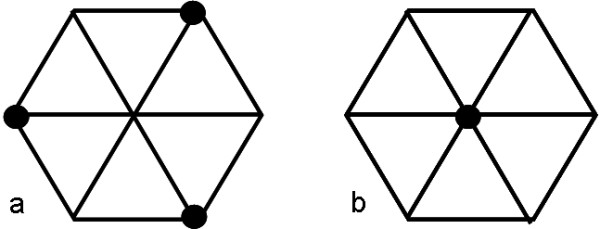
Examples of dominating sets in a graph. Vertices covered by black discs form the dominating set.

This approach begs the question how one can identify and select these structures that are representative of large protein families. In general, target selection for structural genomics is governed by the principle of trying to maximize the information from a selected target [3]. To this effect a graph can be constructed whose vertices are proteins and edges are placed between vertices whenever the sequence similarity between them is such that comparative modeling can provide a model of adequate accuracy. A set of structures, from which models can be generated for all members of the proteins forming the graph, will correspond to a vertex set with the property that for all other vertices in the graph there is an edge connecting it to a member of this vertex set. Such a set is called a dominating set – an example is shown on Figure 1a. While the determination of the smallest dominating set is considered among the so-called 'hard' problems in computer science [4], it has been suggested that the so-called greedy algorithm (that keeps picking the node with the most neighbors) is nearly optimal [5]. On the graph shown on Figure 1a the greedy algorithm would have chosen an other dominating set, shown on Figure 1b, that consists of a single vertex – clearly an optimal choice. For the problem of covering the whole protein space, it has been shown that the greedy algorithm is two to three-fold more efficient than selecting structures randomly and in an uncoordinated fashion [2].

This situation is, however, complicated by the fact that new proteins are continuously added to a set of unknowns, and the target selection is not guided exclusively by the aim of optimizing the coverage of a protein space. As such, there is no practical way to realize the greedy algorithm. However, if the proteins with no experimental structures are given a weight representing their importance for the process of covering the protein space, then the approach inherent in the greedy algorithm can be reformulated to selecting proteins with probability proportional to their weight. Such approach may yield an efficiency that is close to that of the greedy algorithm while providing additional flexibility in the target selection for experimental structure determination. This weighting is the conceptual basis for Pspace, a new computer program that we are describing in this study.

In addition to providing a guide for the coverage of a protein space, Pspace also has a facility for the search of ortholog candidates.

## Methods

### Strategies for covering a protein space

As discussed above, there may be other algorithms besides the greedy algorithm that afford more freedom without significantly lowering the efficiency of coverage. We implemented into Pspace four different algorithms to select proteins for structure determination in order to be able to assess their relative efficiency. Upon selection of a protein for structure determination each proceeds by updating the weights that represent the respective information content of the rest and stops when only structures with zero weight remain:

**1**. Greedy and coordinated: Determine structures of proteins with the highest weights in the set *U*.

**2**. Stochastic and coordinated: Determine structures of proteins from the set *U *with a probability proportional to a weight associated with each protein.

**3**. Random and coordinated: Determine structures of proteins from the set *U *with uniform probability considering only proteins whose weight is positive.

**4**. Random and uncoordinated: Determine structures of proteins from the set *U *with uniform probability considering all proteins in the set *U*.

Vitkup et al [2] showed that for the entire protein space the random and uncoordinated approach requires 2–3 times more structure determination than the greedy algorithm.

### General formalism for calculation of structural information

**Figure 2:**
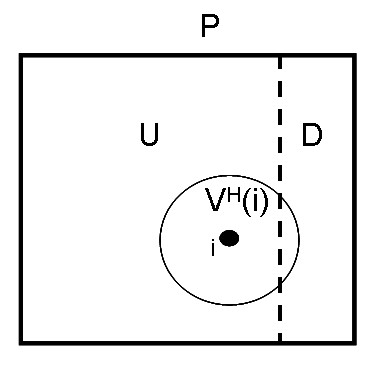
Schematics of the relation between the sets *P, U, D*, and *V*^*H*^(*i*).

At any given time, let *P *be the set of all proteins with known sequences, *D *be the set of all proteins with known sequences and structures, and *U *= *P*\*D*, the set of proteins with known sequence but unknown structure. For a protein *i*, let's define its 'sequence vicinity', *V*^*H*^(*i*), as the set of proteins with unknown structure that are close to *i *in the protein space, i.e., their measure of similarity exceeds a threshold:

*V*^*H*^(*i*) = {*j *| *h*_*ij *_> *H*}

where *h*_*ij *_is a similarity measure in the protein space (e.g., percent of sequence identity) and *H *is the threshold value below which structure determination with comparative modeling is considered unreliable – see Figure 2. For the sequence identity as a measure, 30% has been suggested as a reasonable choice [2].

Assume further, that there is a function *I*(*i*, *S*), giving the amount of information one can gain from knowledge of structures of the proteins in the set *S *about the structure of the protein *i*. Then the utility of determining the structure of a protein *k*, *k *∈ *U*, is the total amount of information gained when protein *k *is added to the set *D*. This utility can be expressed as:

ΔI(k)=I(i,{D,k})−I(i,D))i∈VH(k)
 MathType@MTEF@5@5@+=feaafiart1ev1aaatCvAUfKttLearuWrP9MDH5MBPbIqV92AaeXatLxBI9gBaebbnrfifHhDYfgasaacH8akY=wiFfYdH8Gipec8Eeeu0xXdbba9frFj0=OqFfea0dXdd9vqai=hGuQ8kuc9pgc9s8qqaq=dirpe0xb9q8qiLsFr0=vr0=vr0dc8meaabaqaciaacaGaaeqabaqabeGadaaakeaafaqaaeGadaaabaGaeuiLdqKaemysaKKaeiikaGIaem4AaSMaeiykaKcabaGaeyypa0dabaGaemysaKKaeiikaGIaemyAaKMaeiilaWIaei4EaSNaemiraqKaeiilaWIaem4AaSMaeiyFa0NaeiykaKIaeyOeI0IaemysaKKaeiikaGIaemyAaKMaeiilaWIaemiraqKaeiykaKIaeiykaKcabaaabaaabaGaemyAaKMaeyicI4SaemOvay1aaWbaaSqabeaacqWGibasaaGccqGGOaakcqWGRbWAcqGGPaqkaaaaaa@4F03@

Thus, given *P*, *D*, the vicinities *V*^*H *^(*i*), for each sequence *i *and assuming a reasonable form for *I*(*i*, S), Δ*I*(*k*) can be calculated and used as a measure of the importance of obtaining the structure of protein *k*.

Clearly, for *k *∈*D*, Δ*I*(*k*) = 0. Furthermore, the paradigm put forth above suggests that the knowledge of a structure *i *provides information about the structure of all proteins in its sequence vicinity *V*^*H*^(*i*). Also, the amount of information will be assumed to be proportional to the number of residues of the protein(s) whose structure can be obtained by homology modeling. Formally, this can be written as

I0(k,S)={nrk,hkmax⁡≥H0,hkmax⁡<H
 MathType@MTEF@5@5@+=feaafiart1ev1aaatCvAUfKttLearuWrP9MDH5MBPbIqV92AaeXatLxBI9gBaebbnrfifHhDYfgasaacH8akY=wiFfYdH8Gipec8Eeeu0xXdbba9frFj0=OqFfea0dXdd9vqai=hGuQ8kuc9pgc9s8qqaq=dirpe0xb9q8qiLsFr0=vr0=vr0dc8meaabaqaciaacaGaaeqabaqabeGadaaakeaacqWGjbqsdaWgaaWcbaGaeGimaadabeaakiabcIcaOiabdUgaRjabcYcaSiabdofatjabcMcaPiabg2da9maaceqabaqbaeaabiGaaaqaaiabd6gaUjabdkhaYnaaBaaaleaacqWGRbWAaeqaaOGaeiilaWcabaGaemiAaG2aa0baaSqaaiabdUgaRbqaaiGbc2gaTjabcggaHjabcIha4baakiabgwMiZkabdIeaibqaaiabicdaWiabcYcaSaqaaiabdIgaOnaaDaaaleaacqWGRbWAaeaacyGGTbqBcqGGHbqycqGG4baEaaGccqGH8aapcqWGibasaaaacaGL7baaaaa@5076@

where *nr*_*k *_is the number of residues in *k *and

hkmax⁡=max⁡i∈Shi,k
 MathType@MTEF@5@5@+=feaafiart1ev1aaatCvAUfKttLearuWrP9MDH5MBPbIqV92AaeXatLxBI9gBaebbnrfifHhDYfgasaacH8akY=wiFfYdH8Gipec8Eeeu0xXdbba9frFj0=OqFfea0dXdd9vqai=hGuQ8kuc9pgc9s8qqaq=dirpe0xb9q8qiLsFr0=vr0=vr0dc8meaabaqaciaacaGaaeqabaqabeGadaaakeaacqWGObaAdaqhaaWcbaGaem4AaSgabaGagiyBa0MaeiyyaeMaeiiEaGhaaOGaeyypa0ZaaCbeaeaacyGGTbqBcqGGHbqycqGG4baEaSqaaiabdMgaPjabgIGiolabdofatbqabaGccqWGObaAdaWgaaWcbaGaemyAaKMaeiilaWIaem4AaSgabeaaaaa@425D@

i.e., the similarity between *k *and the protein in the set *S *most similar to it.

### Establishing the similarity measure *h*_*ij*_

The two most common measures of sequence similarity between two proteins are the percent of identical residues and the alignment score – the latter being a function of the similarity matrix and gap penalties used in the alignment. The alignment score can be normalized by the maximum possible score to make it commensurate with the percent identity. Pspace considers both measures in a combined manner:

*I*_0_(*k*, *S*) = min {*I*^*p*^_0_(*k*, *S*), *I*^*s*^_0_(*k*, *S*)}

Where the superscripts *p *and *s *refer to using the percent identity or similarity score, respectively, in Equation 3. The percent identity between two sequences is calculated relative to the number of residues in the shorter sequence. In this treatment, *I*_0_(*k*, *S*) will be nonzero only when both measures fall above their respective thresholds. Recent work quantified the accuracy of homology models [6] as a function of sequence similarity and their result can be used in selecting the threshold values used by Pspace.

The measures discussed above treat all residues equally. Proteins, however, usually have a selected set of residues that are directly involved in the protein's function. For any single protein, the residues forming the binding site may be known. For a family of proteins, conserved residues are usually assumed to have special roles. It is thus reasonable to assume that for such residues higher level of similarity is required than for the rest. Pspace allows the specification of such a selected set with corresponding thresholds that can be different from the thresholds used for the rest of the residues.

### Higher order approximations to the utility function *I*(*k*, *S*)

The zeroth order approximation to *I*(*i*, S) described by Equation 3 above is based on a discretized representation of sequence similarity. In general, however, the amount and reliability of information regarding the structure of protein *k *that can be obtained from the knowledge of a set of proteins *S *a continuous function of the extent of similarities between *k *and the members of the set *S*, {*h*_*i*, *k*_|*i *= 1 ,..., |*S*|} (|*S*| is the number of elements in the set *S*). Besides increased sequence similarity, the reliability of a homology model for *k *derived from the set *S *increases with the number of proteins with significant similarity to the protein *k *in question. Since all these effects are ignored in writing Equations 3 and 4 we present here two more general forms of *I*(*i*, S).

At the next level of approximation the step function used for each measure could be replaced by a sigmoidal function *p*(*h*) multiplied by the number of residues *nr*_*k*_:

*I*_1_(*k*, *S*) = *nr*_k* _*p*(*h*_*k*_^max^)

where *p*(*h*) is zero below the threshold value and gets close to one for *h*>*H *and reaches one at the measure of perfect similarity (i.e., identity). Its actual form can be established by the study of a large set of models whose accuracy is reasonably well known – the work of Chakravarty *et al*., will be useful for this purpose as well [6].

When multiple measures are used – such as in Pspace – then *I*_1_(*k*, *S*) (as well as the further generalizations described below) can be obtained as a weighted average:

*I*_1_(*k*, *S*) = ∑*w*_*k*_*I*^*k*^_1_(*k*, *S*)

where the superscript *k *indicates one of the measures and the *w*_*k*_'s sum up to one.

The effect of using more than one structure for the homology-based estimation of the structure of the protein *k *can be incorporated in the first approximation by multiplying *I*_1_(*k*, *S*) with a function *f*_N_(|*S*|, *p*), representing the additional information the multiple reference structures represent:

*I*_2_(*k*, *S*) = *f*_N_(|*S*|, *p*(*h*_*k*_^max^)) *I_1_(*k*, *S*).

Clearly, *f*_N_(1, *p*) = 1.*f*_N_(|*S*|, *p*) should be monotonically increasing as a function of |*S*| but level off at a value under 1/*p *when |*S*| reaches the number of structures that was found to be sufficient to accurately determine an unknown protein's structure.

At higher levels of approximation, instead of relying on just the homology to the nearest structure, a weighted sum of all *p*(*h*_*i*, *k*_)*nr_*i*, *k *_could be used:

I3(k,S)=∑w(i,k)j∈S nri,k∗p(hi,k).
 MathType@MTEF@5@5@+=feaafiart1ev1aaatCvAUfKttLearuWrP9MDH5MBPbIqV92AaeXatLxBI9gBaebbnrfifHhDYfgasaacH8akY=wiFfYdH8Gipec8Eeeu0xXdbba9frFj0=OqFfea0dXdd9vqai=hGuQ8kuc9pgc9s8qqaq=dirpe0xb9q8qiLsFr0=vr0=vr0dc8meaabaqaciaacaGaaeqabaqabeGadaaakeaacqWGjbqsdaWgaaWcbaGaeG4mamdabeaakiabcIcaOiabdUgaRjabcYcaSiabdofatjabcMcaPiabg2da9maaqaeabaWaaCbeaeaacqWG3bWDcqGGOaakcqWGPbqAcqGGSaalcqWGRbWAcqGGPaqkaSqaaiabdQgaQjabgIGiolabdofatbqabaaabeqab0GaeyyeIuoakiabbccaGiabd6gaUjabdkhaYnaaBaaaleaacqWGPbqAcqGGSaalcqWGRbWAaeqaaOGaey4fIOIaemiCaaNaeiikaGIaemiAaG2aaSbaaSqaaiabdMgaPjabcYcaSiabdUgaRbqabaGccqGGPaqkcqGGUaGlaaa@53B6@

*j *∈ *S*

A logical first choice for the weights *w*(*i*, *k*) would be *f*_N_(|*S *∩ *V*^*D*, l^|)/|*S *∩ *V*^*D*, l^| since for the case when all *h*_*i*, *k*_'s are the same it results in *I*_3_(*k*, *S*) = *I*_2_(*k*, *S*).

### Updating *I*(*k*, *S*) with new sequences and/or structures

When a sequence *k *is added to the set *U *(i.e., the structure is unknown) then we need to calculate the amount of information its structure would yield, Δ*I*(*k*). This calculation requires the determination of its neighborhood. When the structure corresponding to a known sequence is determined (i.e., moved from the set *U *to the set *D*) then all of the Δ*I*(*k*) values of sequences in its vicinity have to be updated. Since the set *U *is in general quite large, the algorithms for these updates have to be considered carefully.

Adding a new sequence to *U *requires the alignment of this sequence with all members of the sets *U *and *D*. Given the large number of sequences already determined and its nearly exponential growth this is in itself a major task. It has been addressed by several groups. Most recently, a database of similarity scores of all known proteins was made available that is now continually being updated as new sequences are being determined [7].

However, for our purpose the results should only be stored for those protein pairs that are within the threshold of utility. The alignment results will give directly *I*(*i*, S) for the new protein. We have to update the *I*(*i*, S) values 'just' for the proteins in the set *U *that have high enough sequence similarity to it that adding *i *to their neighborhood will increase their *I*(*i*, S). This results in a limited number of updates. Finally, the sum of weights derived from the *I*(*i*, S)'s have also to be updated if the weights have to be turned into probabilities for the sampling algorithm.

### Ortholog search

When proteins (or clusters of proteins) in two sets representing proteins in two organisms can be paired by mutual relation of maximum similarity, then the determination of orthologs is straightforward [8]. Pspace, however, is prepared to treat cases when this is not necessarily the case: it establishes a match between the two sets with the best *overall *similarity. For this calculation, the so-called Hungarian method of graph theory [9] is used that establishes the match between two sets that maximizes the overall similarity. In any event, the result of such matching needs further verification based on the biological roles of the proteins matched. The methods for detecting orthologs in distant families (where the seqence similarity of orthologs can be quite low) has recently been reviewed by Wan and Xu [10].

## Results

### Comparison of coverage strategies

**Figure 3:**
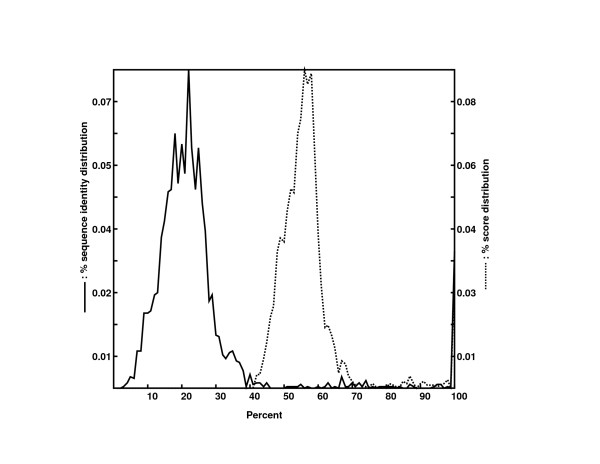
Full line: distribution of pair-wise percent identity for the sequences in the human bromodomain set; Dotted line: distribution of the pair-wise score percentage (the score for a perfect match represents 100%) for the sequences in the human bromodomain set.

Pspace was tested on the sequences in the bromodomain family, as extracted from the SmartEMBL [11] database. Specifically, we selected the bromodomains from proteins in yeast, rat, mouse and humans. For protein alignments we used the PAM-120 scoring matrix, extracted from the database AAindex, Version 3.0 [12,13]. The initial gap penalty was set to 12 and the gap extension penalty was set to 1. The distribution of the percent identities and alignment score percentages in the human bromodomain set are shown on Figure 3 as calculated by Pspace.

The input sequences were checked for redundancy by clustering at near-identity level, using a hierarchical clustering based on minimal cluster member distance [14]. This resulted in a significant reduction in the number of sequences. In the human bromodomain family, clustering at the 99.0, 99.5, or 99.9% similarity level reduces 248 sequences to 65, 70, and 79 sequences, respectively. No redundancy was found at the 100% level, indicating that there was some difference between any pair in the family. The yeast bromodomain set was reduced from 16 to 14 at the 99% similarity level. On the other hand, neither the rat nor the mouse bromodomains had redundant members at the 99% level.

We also used the bromodomains of this four sets (after clustering at the 99% level) to compare the four strategies for the covering of the set. After having aligned the sequences, we determined the percent identity and alignment score percentage. For each protein we calculated *I*_0_(*k*, *S*), using uniformly 25% for minimum percent identity and 40% for minimum similarity score. Different powers of *I*_0_(*k*, *S*) were used for the selection weight. Also, the various random and stochastic strategies were executed 10 times with different random number seeds. Table 1 provides the result for the different strategies.

**Table 1:** Results for the different strategies to cover the bromodomain space

Strategy	Weight	Mean number of structure determinations	S.D.
Greedy		35	
Stochastic and coordinated	[*I*_0_(*k*, *S*)]	41.2	2.0
Stochastic and coordinated	[*I*_0_(*k*, *S*)]^2^	38.0	2.7
Stochastic and coordinated	[*I*_0_(*k*, *S*)]^3^	36.4	2.0
Random and coordinated	Const.	44.0	2.64
Random and uncoordinated	Const.	86.8	3.6

These results clearly show that the stochastic and coordinated approach performs close to the greedy algorithm if the weights are 'strong' enough. It is also clear that the large gain in the number of structures needed comes from the coordination. This emphasizes the fact that the most important step in reducing the number of structure determinations is the coordination of efforts.

### Test of the ortholog searches

The search for orthologs was run to match the rat and mouse bromodomains. The matched sequences are shown in Table 2. Most matches found are indeed orthologs. There are several sequences in the set of rat bromodomains whose function is unknown thus the matches found may be considered a prediction for their function. Note also that for matches found that turned out not to be orthologs, there were no true orthologs represented in this sequence database.

**Table 2:** Mutually best matches between rat and mouse bromodomains

Rat bromodomain	Mouse bromodomain	OK?	%score	% id
ENSRNOP00000016581/43–153	ENSMUSP00000061312/43–153	O	99	99
ENSRNOP00000016581/175–289	ENSMUSP00000061312/175–289	O	100	100
ENSRNOP00000016581/379–489	ENSMUSP00000061312/379–489	O	99	99
ENSRNOP00000016581/516–627	ENSMUSP00000061312/516–627	O	100	100
ENSRNOP00000016581/651–765	ENSMUSP00000061312/651–765	O	100	100
ENSRNOP00000016581/775–881	ENSMUSP00000061312/775–881	O	98	97
Q6MGA9_RAT/71–181	Q7JJ13_MOUSE/71–181	O	100	100
Q6MGA9_RAT/345–454	Q7JJ13_MOUSE/345–454	O	100	100
Q75QC6_RAT/111–217	Q3UKS1_MOUSE/111–217	O	100	100
UPI00001813C2/129–237	BRD7_MOUSE/129–237	O	100	100
UPI0000182A85/235–342	ENSMUSP00000073649/279–386	U	91	85
UPI00001D014C/663–771	Q8CFP4_MOUSE/21–129		63	31
UPI00001D0817/1330–1438	UPI0000020C93/1333–1441		98	95
UPI0000250558/625–733	ENSMUSP00000071391/624–732		100	100
UPI0000250E9C/722–830	Q3TZ59_MOUSE/719–827	O	100	100
UPI000025122E/696–800	TIF1B_MOUSE/697–801	O	100	100
UPI0000500889/1036–1142	SMUSP00000033339/1171–1277	U	100	100
UPI0000500ABF/1387–1515	UPI000042B0CA/1391–1501		97	99
UPI0000500BE2/1085–1195	SMUSP00000087991/1085–1195	O	100	100
UPI0000500F2E/1000–1108	Q6P9L3_MOUSE/993–1101	U	99	98
UPI000050107F/1159–1272	UPI0000565734/1156–1267	O	98	98
UPI000050107F/1321–1426	UPI0000565734/1317–1422	O	99	99
UPI00005018FB/24–134	ENSMUSP00000031215/24–134		97	91
UPI00005018FB/268–377	ENSMUSP00000031215/268–377		97	93
UPI0000502201/158–266	UPI00001C60A3/153–261	O	99	98
UPI0000502202/784–892	BRD8_MOUSE/778–886	O	100	100
UPI000050282E/976–1099	UPI00004351D5/972–1095		97	95
UPI00005029F4/667–775	ENSMUSP00000004985/88–196	U	98	97
UPI0000502B77/30–140	Q5CCJ9_MOUSE/31–141	U	100	100
UPI0000502B77/306–415	Q5CCJ9_MOUSE/307–416	U	100	100
UPI0000502F4F/171–278	UPI0000566B4C/171–278	O	100	100
UPI0000503116/900–1005	TIF1A_MOUSE/902–1007		100	100
UPI0000503365/1–73	UPI0000564B4C/1047–1157	U	64	31
UPI0000503A1F/764–872	ENSMUSP00000039757/742–850	O	99	99
UPI0000503C43/1742–1850	UPI0000026135/1734–1842	U	99	99
UPI0000503CEB/56–166	ENSMUSP00000003726/56–166	O	100	100
UPI0000503CEB/351–460	ENSMUSP00000003726/351–460	O	100	100
UPI0000503EC8/965–1077	UPI00001C594E/955–1067		100	100
UPI00005040EC/1422–1533	Q6AXG8_MOUSE/1422–1533	O	100	100
UPI000050419C/1544–1678	UPI0000565818/1535–1669	U	99	98
UPI00005042C5/646–749	SMUSP00000034787/1158–1261	O	100	100
UPI00005042C5/806–911	SMUSP00000034787/1318–1423	O	99	99
UPI00005045CC/670–773	UPI000056580C/258–361	O	97	92
UPI00005049DA/1399–1507	Q80UV9_MOUSE/799–907	O	100	100
UPI00005049DA/1521–1630	Q80UV9_MOUSE/921–1030	O	99	99

O or U in the column headed with OK? marks matches that are actual orthologs and where the function of the rat sequence is unknown, respectively. The columns headed by %score and %id give the measure of similarity between the matched sequences as the percentage of the maximum possible score and of the identical residues, respectively.

We also compared the yeast and human sets remained (after clustering at the 99% level). While the 14 yeast bromodomains were unequivocally matched to counterparts in the human bromodomain set, none of the matches found corresponded to actual orthologs. This is not surprising since yeast and humans are very distant in the evolutionary tree. This raises the question of how close the score between true orthologs are to the score of the best match. This can be tested by asking Pspace to list for each yeast bromodomain all human bromodomains whose matching score is within a certain percent of the best score. For example, the score of the human ortholog of the GCN5 is within 5% of the best score, but so are 6 other bromodomains.

## Discussion

### Potential scope

Since structures of individual domains of complex multi-domain proteins are often determined separately (as is the case for the bromodomains discussed here) the current implementation of Pspace is best suited for the treatment of a specific family or a limited set of families. However, the concept of dynamically assigning a weight to proteins with unknown structure is applicable to the space of all proteins and can be a valuable help in selecting proteins for structure determination. This can be achieved by implementing the calculation of these weights on a web-based server. Since the results of this study clearly showed that the major gain in the efficiency of covering a protein space comes from coordination, the effect of creating such a server would be a significant gain in the efficiency of covering the protein space. Note that effort of this scale has already been undertaken: the SIMAP server [15] provides the dynamically updated similarity matrix of all known protein sequences.

### Multi-domain proteins

A large number of eukaryotic proteins consist of multiple domains, each of which can be homologous to different domains of other proteins. This has led to the conclusion by Liu and Rost [16] that *'there is no reasonable way of classifying proteins without dissecting proteins into structural domain-like fragments'*. Thus the current capabilities of Pspace limit it to the treatment of single-domain proteins. The formalism developed here, however, can be readily extended to multiple domain proteins by separating the proteins in the set *D *into its domains and calculating the information content of each separately. The introduction of *nr*_*i*, *k *_into the formalism allows the proper account of the domains' contribution. Fortunately, the decomposition is only necessary for the proteins in the set *D *since the knowledge of the structure allows, reasonably, reliable establishment of domain boundaries.

## Conclusion

Pspace is available at the URL . The distribution includes the source code, the matrices of the AAindex database and the (HTML) documentation. A list of its currently implemented functions is given in the Appendix.

## Competing interests

The author(s) declare that they have no competing interests.

## Authors' contributions

The project was jointly designed by both authors. MM developed the algorithms and wrote the software.

## Appendix: List of functionalities of Pspace

The current version of Pspace performs a following set of functionalities:

1. Select and read a scoring matrix. The 66 matrices provided by the database AAindex, Version 3.0 [12] have been included into the distribution [13].

2. Read a set of sequences (in FASTA format) either as first or as second set (for possible ortholog search). Upon reading a set Pspace generates a Postscript plot of the distribution of pairwise % identities and alignment scores within the set.

3. Cluster the sequences in a set by % homology and select the one in the 'middle' as the representative of the cluster. The 'middle' is defined as the sequence whose lowest homology with the rest of the cluster members is the highest.

4. Initialize the weight calculation, assuming that no structure has been determined for the proteins represented by the sequences in the set.

5. Add sequences representing proteins with known structure to a set.

6. Add sequences representing proteins with unknown structure to a set.

7. Find a subset of sequences that covers the whole set using one of the four algorithms described above.

8. Match the sequences on the two sets using one of the three optimization procedures described above.

9. Report the weights assigned to the sequences in a set.

10. Report the content of the whole database (sequences, pairwise scores, weights).

11. Save the database.

12. Restore the database.
